# A Simple and Novel Strategy for the Production of a Pan-specific Antiserum against Elapid Snakes of Asia

**DOI:** 10.1371/journal.pntd.0004565

**Published:** 2016-04-08

**Authors:** Kavi Ratanabanangkoon, Kae Yi Tan, Sukanya Eursakun, Choo Hock Tan, Pavinee Simsiriwong, Teeraporn Pamornsakda, Witthawat Wiriyarat, Chaiya Klinpayom, Nget Hong Tan

**Affiliations:** 1 Laboratory of Immunology, Chulabhorn Research Institute, Bangkok, Thailand; 2 Chulabhorn Graduate Institute, Bangkok, Thailand; 3 Department of Molecular Medicine, University of Malaya, Kuala Lumpur, Malaysia; 4 Department of Pharmacology, Faculty of Medicine, University of Malaya, Kuala Lumpur, Malaysia; 5 Department of Pre-clinic and Applied Animal Science, Faculty of Veterinary Science, Mahidol University, Salaya, NakornPrathom, Thailand; 6 The Veterinary and Remount Department, The Royal Thai Army, NakornPrathom, Thailand; Universidad de Costa Rica, COSTA RICA

## Abstract

Snakebite envenomation is a serious medical problem in many tropical developing countries and was considered by WHO as a neglected tropical disease. Antivenom (AV), the rational and most effective treatment modality, is either unaffordable and/or unavailable in many affected countries. Moreover, each AV is specific to only one (monospecific) or a few (polyspecific) snake venoms. This demands that each country to prepare AV against its local snake venoms, which is often not feasible. Preparation of a ‘pan-specific’ AV against many snakes over a wide geographical area in some countries/regions has not been possible. If a ‘pan-specific’ AV effective against a variety of snakes from many countries could be prepared, it could be produced economically in large volume for use in many countries and save many lives. The aim of this study was to produce a pan-specific antiserum effective against major medically important elapids in Asia. The strategy was to use toxin fractions (TFs) of the venoms in place of crude venoms in order to reduce the number of antigens the horses were exposed to. This enabled inclusion of a greater variety of elapid venoms in the immunogen mix, thus exposing the horse immune system to a diverse repertoire of toxin epitopes, and gave rise to antiserum with wide paraspecificity against elapid venoms. Twelve venom samples from six medically important elapid snakes (4 *Naja spp*. and 2 *Bungarus spp*.) were collected from 12 regions/countries in Asia. Nine of these 12 venoms were ultra-filtered to remove high molecular weight, non-toxic and highly immunogenic proteins. The remaining 3 venoms were not ultra-filtered due to limited amounts available. The 9 toxin fractions (TFs) together with the 3 crude venoms were emulsified in complete Freund’s adjuvant and used to immunize 3 horses using a low dose, low volume, multisite immunization protocol. The horse antisera were assayed by ELISA and by i*n vivo* lethality neutralization in mice. The findings were: a) The 9 TFs were shown to contain all of the venom toxins but were devoid of high MW proteins. When these TFs, together with the 3 crude venoms, were used as the immunogen, satisfactory ELISA antibody titers against homologous/heterologous venoms were obtained. b) The horse antiserum immunologically reacted with and neutralized the lethal effects of both the homologous and the 16 heterologous Asian/African elapid venoms tested. Thus, the use of TFs in place of crude venoms and the inclusion of a variety of elapid venoms in the immunogen mix resulted in antiserum with wide paraspecificity against elapid venoms from distant geographic areas. The antivenom prepared from this antiserum would be expected to be pan-specific and effective in treating envenomations by most elapids in many Asian countries. Due to economies of scale, the antivenom could be produced inexpensively and save many lives. This simple strategy and procedure could be readily adapted for the production of pan-specific antisera against elapids of other continents.

## Author Summary

Antivenom is the most effective treatment modality for snake envenoming. However, they are specific and effective against only one or a few snake venoms. Production of antivenom against many snake species covering a wide geographic area of some countries or regions e.g., Asia and Africa, is not yet possible. This study aimed to use a simple procedure to produce horse antiserum which could neutralize many or all medically important elapid (neurotoxic) snakes (cobras, kraits) of Asia. The venoms of 6 elapid species were obtained from 12 different regions/countries of Asia. Nine of these venoms were ultra-filtered to remove high molecular weight, non-toxic proteins to obtain the toxin fractions (TFs) for use as immunogen and thus enable inclusion of greater variety of elapid venoms. The 9 ‘toxin fractions’ together with the remaining 3 crude venoms were used to immunize 3 horses. The antisera of the horses obtained were shown to neutralize, in mice, the lethal effects of the venoms used in the immunization and 16 other Asian/African elapid venoms not used in the immunization. Thus, the simple strategy could broaden the neutralizing capacity of the resulting antiserum. The antiserum could be processed into antivenom with wide paraspecificity and effective against many elapid snakes of Asia.

## Introduction

Snake envenoming is an important medical problem in various developing countries of Asia and Africa [1, 2]. It has been estimated that at least 1.2 million people are affected annually with about 20,000 deaths [3]; however these figures likely represent merely the tip of the iceberg as a result of poor epidemiological records [4–6]. This serious envenoming problem has led WHO to recognize it as one of the neglected tropical diseases [7], in addition to the long recognized status of snakebite as an occupational hazard and a disease of poverty [2]. Despite this, the provision of global funding and technologies aimed at solving the global snakebite envenomation problem has been limited. Moreover, efforts to solve the problem have largely been taken up by regional toxicologists through research initiatives designed to gain a better understanding of the compositional variation of venoms and facilitate the production of effective, affordable, broad-spectrum antivenoms [8, 9]. Indeed, the rational and most effective treatment for snake envenomation is the administration of specific antivenom which remains unavailable in many parts of the world. Fortunately, attempts are being made on various research fronts to produce these antivenoms [9, 10].

Antivenoms (AVs) are usually produced in horses, although other large animals like camels, donkeys and sheep can also be used [11–14]. AV can be monospecific or polyspecific. Monospecific AV is specific and effective against the snake venom used as the immunogen and some related cross-reacting species. Therefore, identification of the culprit snake is necessary. Polyspecific AVs are effective against the multiple venoms that are used in the immunization and some other cross-reacting venoms. The cross-reactivity or paraspecificity of polyspecific antivenom is an important property useful to pan-specific antivenom production. However, one problem with the production of polyspecific AVs is that only about 3–6 snake venoms can be used in the immunization [15, 16]. Although the maximum number of venoms has not been established and reported, it is believed that immunization of more than 5–6 venoms resulted in lower potency of the polyspecific AVs. Thus, in order to increase coverage, 2 (or more) polyspecific AVs were often combined to yield a mixed-polyspecific AV that is effective against a broader spectrum of venoms [16]. Although the mixed-polyspecific AVs are very useful against a wide range of venoms and make identification of the culprit snake prior to AV administration unnecessary, it is probably more expensive to produce since 2 or more groups of horses are needed.

In order to prepare a truly polyspecific AV with wider venom coverage, various technologies have been employed. For example, antivenomics studies of a number of polyspecific AV have identified which of the heterologous venom’s lethal toxins interact, or fail to interact, with the AV antibodies [17–19]. From such studies, those venom toxin antigens that interact weakly or fail to interact with the AV antibody can be isolated and added to the immunogen mix to improve the coverage of the AV. Antivenomics studies could also provide valuable information about common venom antigens and aid in the selection of appropriate venom antigens to be included in the immunogen mix.

Another interesting approach is the use of DNA immunogens termed ‘epitope strings’ [20]. DNA immunization of mice with the ‘epitope string’ resulted in antibodies that could neutralize the toxins of several species of African viper [20]. This technology could be a useful possibility in the future.

These very interesting approaches are promising and could eventually result in the production of effective pan-specific antivenoms. Meanwhile, some simple, readily applicable protocols that would result in effective antivenoms would be desirable.

Snake venoms contain mixtures of more than 100 proteins with different molecular weights and biological activities [21]. Bites by elapids, such as cobras, kraits and mambas, are considered ‘neurotoxic’ as they cause neuromuscular paralysis mediated by low molecular weight toxins of the 3 finger toxin family (3FTs) [22]. The most important toxins among the 3FTs are the postsynaptic neurotoxins (PSNT). PSNTs bind specifically, and quasi-irreversibly, to nicotinic acetylcholine receptors (nAchR) at the neuromuscular junction [23, 24]. Toxin binding results in inhibition of neuromuscular transmission, muscle paralysis and death by respiratory failure [23, 25]. Another important subtype of the 3FTs is the cytotoxins (cardiotoxins), which have cytolytic activity and are mainly involved in local tissue necrosis [26, 27].

In addition to 3FTs, the krait venoms (genus *Bungarus*) also contain lethal basic phospholipase A_2_ presynaptic neurotoxins. These toxins have MWs of about 21–30 kDa [28]. They can cause damage to motor neuron terminals and cause release of the neurotransmitter acetylcholine, from the nerve endings [29]. Depletion of acetylcholine in the nerve terminals results in neuromuscular transmission blockage with death occurring as a result of respiratory failure.

These postsynaptic and presynaptic neurotoxins, as well as acidic phospholipases, are therefore the most important cause of death resulting from elapid envenoming. AVs must be able to neutralize these toxins in order to be effective and life-saving. The other high MW and thus highly immunogenic elapid proteins, mostly hydrolytic enzymes are usually not lethal [30]; antibodies to neutralize these venom proteins are not essential in saving the lives of victims.

From the above information, it should be possible to prepare AVs effective against elapid venoms using only the 3FTs and presynaptic neurotoxins as immunogens. This approach would have the advantage of reducing the total number of venom antigens the horse is exposed to. Consequently, it should be possible to significantly increase the number of different snake venoms used in the immunogen mix. This will expose the horse immune system to a wide variety of lethal toxins from numerous venoms with diverse repertoires of toxin epitopes. This, in turn, should broaden the paraspecificity of the AV antibodies and increase the cross-neutralization of the AV against venoms of species not included in the immunogen mix, hence extending its use to more countries, particularly those which are socioeconomically disadvantaged.

The rationale described above suggests that it should be possible to prepare a ‘pan-specific’ AV effective against most of the elapids of Asia. In the present study, a mixture of ‘toxin fractions’ (TFs) and venoms from 6 medically important elapid venoms (WHO category 1) obtained from 12 regions/countries, was used at low doses to immunize horses. It was shown that the horse antiserum could neutralize all the 27 homologous/heterologous elapid venoms (including 4 African *Najas*) tested. Details of the preparation and characterization of the immunogens and the *in vitro* and *in vivo* potency of the antiserum are described.

## Materials and Methods

### Animals

The horses were of mixed breed and were 3–6 years old and weighed about 420–480 Kg. They were under the care of veterinarians with expertise in equine health at the Animal Hospital of the Animal and Remount Department, The Royal Thai Army, and the veterinarians of the Faculty of Veterinary Science, Mahidol University. There were dewormed to remove gut helminthes and were free of external parasites. The horses were vaccinated against rabies, tetanus, and equine encephalytis. Their hematologic, hepatic and kidney status were tested prior to and monitored during the experiment. They were kept in clean well-ventilated brick-made stables, and were allowed to stay on the pasture for several hours every day. Albino mice (ICR strain, 20-25g) were supplied by the Animal Experimental Unit, Faculty of Medicine, University of Malaya.

### Animal ethics statement

Experiments involving the care, bleeding and immunization of horses with various venoms were reviewed and approved by the Animal Care and Use Committee of the Faculty of Veterinary Science, Mahidol University, Protocol and clearance no.MUVS-2012-69 in accordance with the Guidelines of the National Research Council of Thailand.

The protocol of animal study on mice was based on the guidelines given by the Council for International Organizations of Medical Sciences (CIOMS) and was reviewed and approved by the Institutional Animal Care and Use Committee (IACUC) of the University of Malaya (Ethical clearance No. 2014-09-11/PHAR/R/TCH).

### Chemicals and biochemical

#### Chemicals

All reagents were of reagent grade and were obtained from Sigma Chemical Co., St Louis, MO, USA unless indicated otherwise.

#### Venoms and antiserum

Venoms of *Naja kaouthia* (Thailand) and *Bungarus fasciatus* (Thailand) were purchased from Queen Saovabha Memorial Institute (QSMI), Bangkok. Venoms of adult *Bungarus candidus* (Northeast and South Thailand) were from snakes maintained and milked at QSMI. *Naja kaouthia* (Vietnam) was from Prof. Trinh Xuan Kiem. *Naja sputatrix* (Indonesia) venom used for immunization and *Bungarus candidus* (Indonesia) venom were gifts from PT BioFarma, Bandung, Indonesia; *Naja atra* (China) and *Bungarus multicinctus* (China) venoms were purchased from Yiwu City Jiashang Import & Export Co., Ltd., Zhejiang, China. Venoms of *Naja sputatrix* (Indonesia) used for *in vivo* neutralization assay, *Naja siamensis* (Thailand), *Naja philippinensis* (The Philippines), *Naja atra* (Taiwan), *Naja naja* (India), *Naja haje* (Egypt), *Naja nigricollis* (Cameroon), *Naja melanoleuca* (North Cameroon), *Naja nubiae* (Egypt) and *Bungarus multicinctus* (Taiwan) venoms were purchased from Latoxan (Valence, France). Venoms of *Naja sumatrana* (Malaysia), *Naja kaouthia* (Malaysia) and *Ophiophagus hannah* (Malaysia) were milked from adult snakes captured in Malaysia by Dr. Choo Hock Tan and Prof. Dr. Nget Hong Tan. *Naja oxiana* (Pakistan), *Bungarus caeruleus* (Pakistan) and *Bungarus sindanus* (Sindh, Pakistan) venoms were kind gifts from Dr. Naeem Quraishi, Anti-snake Venom and Anti-rabies Serology Laboratory, Nawabshah, Sindh, Pakistan. These venoms were obtained from adult snakes and were lyophilized and stored desiccated at -20 °C except during transportation. *N*. *kaouthia* (Thailand) principal postsynaptic neurotoxin 3 (NTX3) was purified as described by Karlsson et al [31]. Monovalent horse anti-*Naja kaouthia* (Thailand) serum was purchased from QSMI.

### Preparation of ‘Toxin fractions’ (TFs)

The TFs of elapid venoms were prepared by separately dissolving the venom in 100 mM ammonium acetate, pH 5.0 to make 1.0 mg/ml final protein concentration. High molecular weight venom proteins of the *Naja spp*. were removed by filtration of the venom solution through 30 kDa molecular weight cut off (MWCO) ultra-filtration membrane (Amicon) at 14,000 x g for 10 min at 4°C. Venoms of the *Bungarus spp*. were filtered through 50 kDa MWCO ultra-filtration membrane. The volume of the filtrates and the retentates were recorded and their protein contents were assayed. The filtrates were called ‘toxin fraction’ (TF) which were further characterized by one-dimensional SDS-PAGE, RP-HPLC and protein determination. The crude venoms and the TFs were kept frozen at -20 °C until used.

### Characterizations of TFs of various elapid venoms

#### Sodium dodecyl sulfate polyacrylamide gel electrophoresis (SDS-PAGE)

Protein composition of each TF was analyzed by SDS-PAGE. The SDS-PAGE was performed following the method described by Laemmli [32] under non-reducing conditions using 15% (w/v) separating gel. Electrophoresis was performed at 20 mA constant current for 3 h. The gels were stained with Coomassie Brilliant Blue R-250. A Mark12™ Unstained Standard Protein Ladder (Invitrogen, 2.5–200 kDa) was used as the molecular weight standard. The SDS-PAGE gels of the crude venoms and the TFs were scanned (Chemidoc-It^2^, UVP, LLC, Canada) and the band was measured by image J software (version 1.49, [33]).

#### Separation of proteins of crude venom and TF by reverse phase-HPLC

Venoms or TFs of *Naja kaouthia* (Thailand) (1.5 mg in distilled water) was subjected to C18 (0.4 cm x 25 cm, 5 mm particle size, 300°A pore size) RP-HPLC column (Waters-Micromass, MA, USA) attached to a High Pressure Gradient System coupled with photodiode array detector and micro-auto sampler. The column was eluted at 1 ml/min with a linear gradient of 0.1% trifluoroacetic acid in water (solvent A) and 100% acetonitrile (solvent B) (0–5% B for 10 min, followed by 5–25% B over 20 min, 25–45% B over 120 min, 45–100% B over 5 min, and 100% B over 20 min). The eluted proteins were detected at OD 215 nm and OD 280 nm. The protein fractions of each peak were collected manually and pooled. The proteins fractions were reconstituted in ultrapure water and subjected to 15% SDS-PAGE under denaturing conditions as described above. The band area was measured by image J software (version 1.49, [33]). The proteome of *N*. *kaouthia* TF was determined by comparing the RP-HPLC elution profile of the TF with the RP-HPLC elution profile and proteome of *N*. *kaouthia* (Thailand) venom determined previously [34].

### Immunogen preparation and immunization

#### Immunogen preparation

The immunogens for the immunization of horses were prepared under aseptic conditions as described previously [35]. The TFs of 9 elapids and 3 crude venoms (Table 1) were pooled to give the protein content of each venom/TF used in each immunization as indicated in Table 1. The pooled TFs/crude venoms were then mixed with Freund’s complete adjuvant (or incomplete Freund’s adjuvant) at 1:1 (v/v). The mixture was rigorously homogenized in an Omni Mixer Homogenizer (Serial no.MX21190, Omni International, USA) at 8,750 rpm for 15 min on ice. Alum (Aluminum hydroxide and magnesium hydroxide, lot no.NJ176763) used as adjuvant in the last immunization was prepared as described by the manufacturer (Thermo Scientific). TFs and crude venoms were mixed with Alum at 1:1 (v/v) and homogenized for 15 min on ice. The immunogen was kept on ice until used.

**Table 1:** Elapid crude venoms/toxin fractions used in the immunization of horses.

No	Elapid venom	Source	WHO Category	TF/Crude	LD_50_(μg/g)	Amounts of venom/TF used in immunization (μg/horse)				
						1^st^	2^nd^	3^rd^	4^th^	5^th^
1	*N*.*kaouthia*	Thailand	Cat 1	TF	0.23	25	37.5	75	50	40
2	*N*.*kaouthia*	Malaysia	Cat 1	TF	0.89	25	37.5	75	50	40
3	*N*.*kaouthia*	Vietnam	Cat 1	Crude	0.40	25	37.5	75	50	40
4	*N*.*philippinensis*	Philippines	Cat 1	Crude	0.18	25	37.5	75	50	40
5	*N*.*sputatrix*	Indonesia	Cat 1	TF	0.9	25	37.5	75	50	40
6	*N*.*atra*	China	Cat 1	TF	0.56	25	37.5	75	50	40
7	*N*.*atra*	Taiwan	Cat 1	TF	0.56	25	37.5	75	50	40
8	*B*.*candidus*	Indonesia	Cat 1	TF	0.11	12.5	18.75	37.5	25	20
9	*B*.*candidus*	Thailand (Northeast)	Cat 1	TF	0.11	12.5	18.75	37.5	25	20
10	*B*.*candidus*	Thailand (South)	Cat 1	Crude	0.11	12.5	18.75	37.5	25	20
11	*B*.*multicinctus*	China	Cat 1	TF	0.11	12.5	18.75	37.5	25	20
12	*B*.*multicinctus*	Taiwan	Cat 1	TF	0.11	12.5	18.75	37.5	25	20
Total Venom/TF (μg/horse)						250	375	750	500	340

#### Immunization

Three horses were immunized with the mixture of the 9 TFs and 3 crude venoms at the protein doses (μg/horse) of TFs/venoms shown in Table 1. The immunization was made using the low dose, low volume multi-site immunization protocol [35–37].The immunogen was injected subcutaneously around the horse’s neck at 20 sites (10 injection sites on each side) in a volume of 0.1 ml/site. The details on the immunization and the bleeding schedules together with the types of adjuvants used are shown in Table 2. Alum was used as adjuvant in the last immunization and the number of injection sites was reduced to 6 sites at 0.2 ml/site. Blood samples (150 ml) were collected every 2 weeks from each horse. Sera of each horse were obtained by centrifugation of the clotted blood at 800 x g for 15 min at room temperature. The sera were kept at -20°C until used.

**Table 2:** Details on the immunization of horses.

Week	Immunization	Adjuvant	Number of sites and volume/site	Blood collection
0	1^st^	CFA	20 sites, 0.1ml/site	0 Bleeding (Pre-immunized serum)
2	-	-	-	
3	2^nd^	IFA	20 sites, 0.1ml/site	1^st^Bleeding
4	-	-		
5	3^rd^	IFA	20 sites, 0.1ml/site	2^nd^ Bleeding
6	-	-	-	
7	-	-	-	3^rd^Bleeding
8	-	-	-	
9	-	-	-	4^th^Bleeding
10	-	-	-	
11	-	-	-	5^th^Bleeding
12	4^th^	IFA	20 sites, 0.1ml/site	6^th^Bleeding
13	-	-	-	7^th^Bleeding
14	5^th^	Alum	6 sites, 0.2ml/site	8^th^Bleeding
15	-	-	-	9^th^Bleeding
16	-	-	-	10^th^Bleeding

CFA: complete Freund’s adjuvant; IFA: incomplete Freund’s adjuvant.

### ELISA of specific antibody against the TF of each elapid venom

The antibody titer in each serum sample was determined by an indirect ELISA as described by Rungsiwongse and Ratanabanangkoon [38]. A polyvinyl microtiter plate (Costar) was coated with 50 μl/well of 5 μg/ml (protein content) of each TF in 0.05 M sodium carbonate-bicarbonate buffer pH 9.6 for 18 hr at 4°C. The plate was washed 4 times with 0.05% Tween-20 in normal saline (NSST). The serum from each horse was 4-fold diluted, starting from 1: 100 to 1: 2.6 x 10^7^ in diluting buffer (0.15 M PBS, pH 7.4 containing 0.05% Tween-20 and 0.5% BSA). Then, 50 μl of the sera at various dilutions were added into each well and incubated for 1 hr at room temperature. The wells were washed 4 times with NSST before 50 μl/well of 1: 40,000 diluted sheep anti-horse IgG-horseradish peroxidase conjugate (Sigma) in diluting buffer was added and incubated at room temperature for 1 hr. After 4 washes, 100 μl/well of freshly prepared substrate solution (0.01% 3,3′,5,5′-Tetramethylbenzidine (TMB) and 0.03% hydrogen peroxide in 0.075 M citrate-phosphate buffer, pH 5.0) was added into each well and incubated in the dark for 15 min at room temperature. The reaction was terminated by adding 25 μl of 4 N sulfuric acid. The plates were read at 450 nm against blank using an ELISA reader (Labsystem Multiskan, Ascent). The highest dilution giving an absorbance reading of 0.4 was regarded as the end point titer using GraphPad Prism 6 program. A positive reference serum (pooled monospecific anti-*N*. *kaouthia* horse serum at dilution 1:6400) and the principal postsynaptic neurotoxin (NTX3) of *N*. *kaouthia* used as a standard reference antigen, were added in every plate to correct for day-to-day and plate-to-plate variations.

### Lethality of elapid venoms and the neutralization activity of the ‘pan-specific’ antiserum (pAS)

#### Determination of venom lethality

The median lethal dose (LD_50_) of the venoms were determined by intravenous (*i*.*v*) injection into ICR mice (20–25 g, n = 4 per dose). The survival ratio was recorded after 48 h and LD_50_ was calculated using Probit analysis method [39].

#### Neutralization of elapid venom lethality by the ‘pan-specific’ antiserum (pAS)

Neutralization of venom lethality was experimented as adapted from Ramos-Cerrillo et al. [16]. Equal volumes of the sera of the 3 horses from the 10^th^ bleeding were pooled and used in the *in vivo* neutralization assay; this pooled serum was called ‘pan-specific’ antiserum (pAS). A challenge dose (5 x LD_50_ or lower if pAS failed to cross-neutralize 5x LD_50_) of the venom in 50 μl saline was pre-incubated at 37°C for 30 min with various dilutions of the reconstituted pAS in normal saline, to give a total volume of 250 μl. The mixture was subsequently centrifuged at 10,000 x g for 5 min to eliminate the precipitates before injected intravenously into the caudal vein of the mice (20–25 g, n = 4 per dose). The number of survivors after 48 h was recorded. Principally, the challenge dose used was 5x LD_50_. However, if 200 μl of the reconstituted pAS (maximum amount of antiserum that can be used in mice) failed to give full protection of the mice, a lower venom challenge dose of 2.5x LD_50_ or 1.5x LD_50_ was used instead. Since all the challenge doses either at 5x LD_50_, 2.5x LD_50_ or 1.5x LD_50_ were above LD_100_ of the venoms, control groups of mice were not included to save animal lives. The pAS was considered ineffective when none of the animals injected with the pre-incubated mixture survived. Neutralizing potency of the antiserum was expressed as median effective dose (ED_50_, the amount of reconstituted pAS in μl or the ratio of mg venom/ml reconstituted antiserum that gives 50% survival of the animals tested) or 'neutralization potency' (P, the amount of venom that is completely neutralized by a unit volume of pAS [40]). Potency is theoretcially unaffected by the number of LD_50_ used as challenge.

### Statistical analysis

Median lethal doses (LD_50_) of the venoms and median effective doses (ED_50_) of pAS are expressed with 95% confidence interval (CI). LD_50_, ED_50_ and the 95% CI were calculated using the probit analysis method of Finney [39] with the Biostat 2009 Analysis software (AnalySoft Corp., Bracknell, UK).

### Miscellaneous procedures

Protein concentration was determined as described by Lowry *et al*. [41] using bovine serum albumin as standard. The mass of venoms and other proteins referred to in this study was in protein content as assayed by Lowry et al [41].

## Results

### Preparations and characterizations of the toxin fractions (TFs) from various elapid venoms

**Fig 1:**
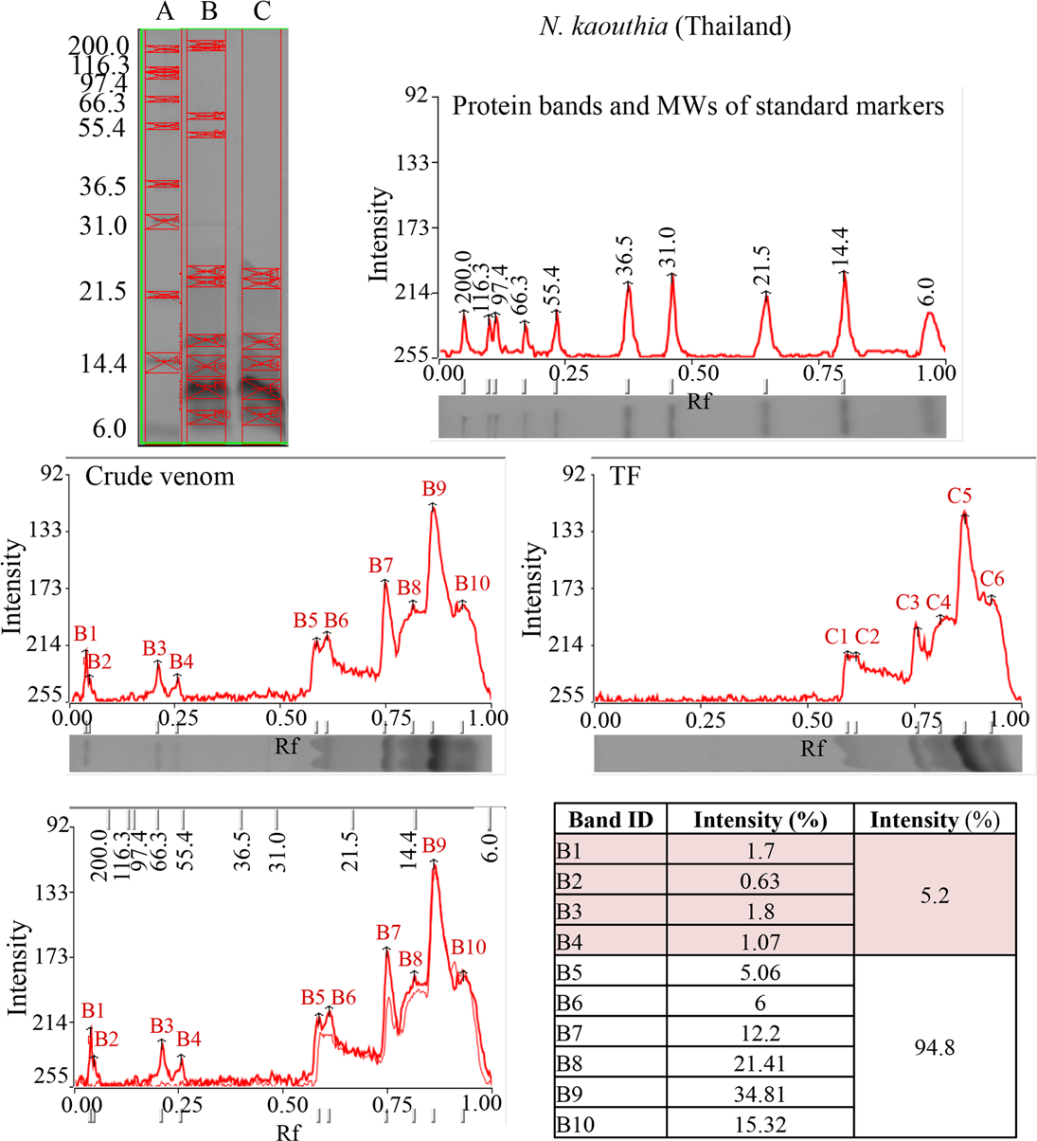
SDS-PAGE of standard protein markers (Lane A), crude venom (Lane B) and TF (Lane C) of *N*. *kaouthia* (Thailand) venom. The scanning of each gel lane is shown together with the relative protein abundance estimated from the intensity of the band.

When the individual venoms of the *Naja spp*. were ultra-filtered through the 30 kD MWCO (molecular weight cut-off) membrane and the *Bungarus spp*. venoms through the 50 kD MWCO filters, the filtrates containing the toxin fractions (TFs) of the venoms were obtained. Fig 1 shows the SDS-PAGE of the crude *N*. *kaouthia* (Thailand) venoms and the corresponding TF. Both the gels and the scanning profiles showed that the high molecular weight proteins (>30 kDa, including protein bands B1, B2, B3 and B4, total 5.2% of protein content by protein assay) were effectively removed while the low MW toxic proteins (proteins B5 to B10) were all present (as C1 to C6, total 94.8% of protein). Similar findings were obtained for the other venoms and their corresponding TFs. The SDS-PAGE and the amount of high molecular weight proteins removed by ultra-filtration are shown in Table 3 and S1 Fig. The protein content of high MW venom proteins removed by the ultrafiltration process range from 3.13% (*B*. *multicinctus*, Taiwan) to 17.52% (*N*. *kaouthia*, Vietnam).

**Table 3:** Percentage of high MW venom proteins removed by ultrafiltration.

No.	Species	High MW proteins removed (%)
1	*N*. *kaouthia* (Thailand)	5.2
2	*N*. *kaouthia* (Malaysia)	14.68
3	*N*. *kaouthia* (Vietnam)	17.52
4	*N*. *sputatrix* (Indonesia)	10.18
5	*N*. *atra* (China)	9.27
6	*N*. *atra* (Taiwan)	16.06
7	*B*. *candidus* (Indonesia)	6.64
8	*B*. *candidus* (Northeast Thailand)	13.44
9	*B*. *multicinctus* (China)	4.7
10	*B*. *multicinctus* (Taiwan)	3.13

**Fig 2:**
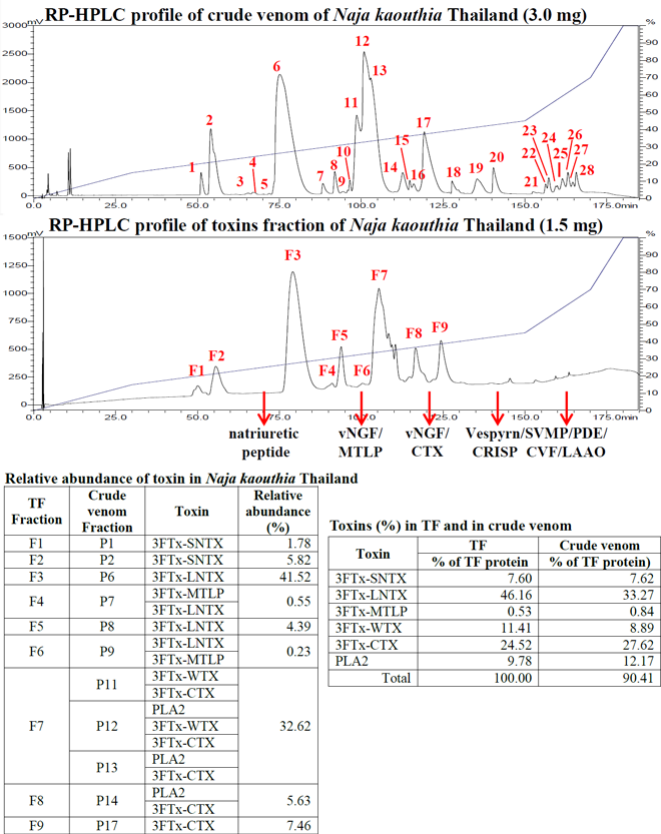
Venomics of *Naja kaouthia* (Vietnam) venom and TF under RP-HPLC.

We also examined the proteome of the TF of *N*. *kaouthia* (Thailand) and compared with the crude venom proteome. TF was subjected to RP-HPLC and the elution profiles are shown in Fig 2. Comparison of the RP-HPLC patterns with that of the crude venom [34] indicated that for *N*. *kaouthia* venom, the ultrafiltration successfully removed the bulk of the high molecular weight venom proteins while retained the lethal, low molecular weight toxins of the venom. All the six groups of venom toxins (short postsynaptic neurotoxin (SNTX), long postsynaptic neurotoxin (LNTX), muscarinic toxin-like proteins (MTLP), weak postsynaptic neurotoxin (WTX), cytotoxin (CTX) and phospholipase A_2_ (PLA_2_) were almost fully retained in the TF whereas the high molecular weight snake venom metalloproteinase (SVMP) and L- amino acid oxidase (LAAO) were totally removed. Table 4 shows the lethality of TFs and venoms from four representative venoms: *N*. *kaouthia* (Thailand and Malaysia), *B*. *candidus* (Northeast Thailand) and *B*. *multicinctus* (China) in a mouse model. The results show that the crude venoms and the corresponding TFs exhibited statistically comparable values of LD_50_s. Since the amount of high MW proteins removed by ultrafiltration was quite small and the LD_50_ determination was subjected to high biological variations, the expected lower LD_50_ values of the TFs were not observed (except in the case of *N*. *kaouthia* Vietnam). However, as mentioned above, the RP-HPLC and venomics data together indicated that all the major lethal toxins of each venom were indeed recovered in their corresponding TFs.

**Table 4:** Comparison of lethality of crude venoms and toxin fractions (TF) of four elapid snakes.

Venom	LD_50_ (μg/g)	LD_50_ (μg/g)
	Crude venom	TF
*N*. *kaouthia* (Thailand)	0.18 (0.12–0.27)	0.18 (0.17–0.20)
*N*. *kaouthia* (Vietnam)	0.90 (0.59–1.37)	0.75 (0.69–0.82)
*B*. *candidus* (Northeast Thailand)	0.09 (0.06–0.14)	0.09 (0.06–0.14)
*B*. *multicinctus* (China)	0.014 (0.010–0.021)	0.021 (0.019–0.024)

### *In vitro* and *in vivo* activities of the horse ‘pan-specific’ antiserum (pAS)

#### ELISA titers of pAS against TFs of various homologous and heterologous venoms

The kinetics and ELISA antibody titers of the sera of each horse at various bleedings against homologous and heterologous elapid venoms are shown in S2 Fig and S3 Fig, respectively. As was observed previously [36, 37, 42], the antibody titers of all 3 horses against the elapid venoms rose rapidly and reached plateau at about 4^th^-6^th^ week. A summary of the mean ELISA titers of all 3 horses is shown in Tables 5 and 6. Among the homologous venoms, the antibody titers against the *Naja spp*. were comparable and higher than those against the two *Bungarus*. Similar results were obtained with the heterologous venoms; the horse sera showed relatively low titers against the two *Bungarus* (*B*. *caeruleus* and *B*. *sindanus*) and *O*. *hannah* venoms.

**Table 5:** Mean antibody titer of ‘pan-specific’ antiserum (pAS) against homologous elapid venoms.

	Mean Titer (x 10^4^)							
	*N*.*kaouthia* (Thailand)	*N*.*kaouthia* (NTX3)	*N*.*atra* (China)	*N*.*atra* (Taiwan)	*N*.*philippinesis*(Philippines)	*N*.*sputatrix* (Indonesia)	*B*.*candidus*(Indonesia)	*B*.*multicinctus* (China)
Bleeding 0	0.0001 ± 0	0.0001 ± 0	0.0001 ± 0	0.0001 ± 0	0.0001 ± 0	0.0001 ± 0	0.0001 ± 0	0.0001 ± 0
Bleeding 1	3.6 ± 3.4	0.1 ± 0.4	5.0 ± 5.0	4.1 ± 4.6	1.5 ± 2.5	4.8 ± 4.0	0.1 ± 0.3	0.1 ± 0.1
Bleeding 2	38.3 ± 54.1	6.1 ± 9.3	42.5 ± 54.8	36.8 ± 59.1	20.1 ± 30.5	44.6 ± 59.5	12.3 ± 29.2	8.4 ± 29.1
Bleeding 3	59.0 ± 7.0	15.3 ± 3.1	66.0 ± 15.7	47.4 ± 8.4	47.4 ± 9.4	71.8 ± 17.1	20.6 ± 20.2	26.0 ± 28.1
Bleeding 4	52.7 ± 16.9	12.9 ± 1.9	43.3 ± 11.1	36.1 ± 6.4	38.6 ± 11.9	54.1 ± 15.0	16.4 ± 11.8	20.1 ± 15.0
Bleeding 5	48.3 ± 24.3	11.7 ± 2.4	41.2 ± 14.0	45.3 ± 18.3	27.0 ± 12.0	48.7 ± 16.1	12.2 ± 6.5	15.9 ± 9.4
Bleeding 6	41.1 ± 21.4	9.7 ± 1.1	36.6 ± 13.6	38.6 ± 13.8	23.6 ± 13.6	44.7 ± 11.5	11.9 ± 8.4	15.0 ± 6.2
Bleeding 8	46.3 ± 34.6	16.6 ± 7.8	40.3 ± 28.0	43.1 ± 29.8	41.0 ± 24.2	56.0 ± 33.1	19.6 ± 12.6	29.3 ± 19.7
Bleeding 10	60.2 ± 24.6	18.5 ± 5.4	47.5 ± 21.6	46.7 ± 20.4	47.6 ± 17.5	59.1 ± 22.8	24.6 ± 11.0	35.6 ± 16.3

**Table 6:** Mean antibody titer of ‘pan-specific antiserum’ (pAS) against heterologous elapid venoms.

	Mean Titer (x 10^4^)						
	*O*.*hannah* (Thailand)	*N*.*sumatrana* (Malaysia)	*N*.*siamensis* (Thailand)	*N*.*oxiana* (Pakistan)	*N*.*naja* (India)	*B*.*caeruleus*(India)	*B*.*sindanus* (Pakistan)
Bleeding 0	0.0001 ± 0	0.0001 ± 0	0.0001 ± 0	0.0001 ± 0	0.0001 ± 0	0.0001 ± 0	0.0001 ± 0
Bleeding 1	0.0 ± 0.0	2.7 ± 4.0	3.2 ± 3.7	2.2 ± 3.1	2.9 ± 3.4	0.0 ± 0.0	0.0 ± 0.0
Bleeding 2	1.3 ± 3.4	33.9 ± 63.4	31.4 ± 42.4	25.5 ± 35.4	30.3 ± 46.7	3.6 ± 9.5	2.8 ± 6.2
Bleeding 3	4.3 ± 2.8	58.4 ± 13.0	70.0 ± 8.2	56.6 ± 4.0	54.6 ± 10.3	9.0 ± 8.5	11.7 ± 7.9
Bleeding 4	4.8 ± 2.5	55.5 ± 25.6	59.2 ± 8.4	49.4 ± 25.2	44.3 ± 17.9	7.9 ± 5.3	10.9 ± 5.6
Bleeding 5	4.2 ± 2.2	32.8 ± 14.1	40.2 ± 17.1	33.2 ± 17.7	32.1 ± 12.8	6.1 ± 3.8	8.7 ± 4.5
Bleeding 6	3.4 ± 2.0	28.5 ± 13.3	36.3 ± 18.3	27.5 ± 10.1	27.5 ± 9.1	5.0 ± 2.5	7.4 ± 3.6
Bleeding 8	5.4 ± 4.4	32.3 ± 29.0	43.6 ± 31.0	26.6 ± 18.0	26.8 ± 32.1	10.8 ± 6.9	11.3 ± 7.9
Bleeding 10	8.1 ± 2.8	36.9 ± 20.9	59.9 ± 40.1	35.7 ± 15.9	26.2 ± 11.1	16.1 ± 7.7	17.6 ± 7.7

### LD_50_ and ED_50_

The lethal effects measured in median lethal doses (LD_50_s) of all the 27 homologous and heterologous venoms including 4 African elapids are shown in Table 6. The LD_50_s of these snake venoms varied widely. The most lethal venom was that of *B*. *sindanus* (LD_50_ of 0.018 μg/g) while the least toxic (LD_50_ of 1.80 μg/g) was *N*. *naja* (India) venom. In general, the *Bungarus* venoms were more lethal than those of the *Naja*. It should be mentioned that the amount of *B*.*candidus* (South Thailand) venom which was used as immunogen was not enough for the *in vivo* neutralization assay.

The effective doses at 50% survival rate (ED_50_s) of the pAS against the homologous and heterologous venoms are shown in Tables 7 and 8, respectively. Overall, the pAS could neutralize all the venoms tested including 4 African *najas* with different degree of effectiveness. For the homologous venoms, the potency value (P) which is theoretically independent of the challenging dose [40] ranged from 0.712 mg/ml against *N*. *kaouthia* (Malaysia) venom to 0.101 mg/ml against *N*. *kaouthia* (Thailand). Among the heterologous venoms, the potency value (P) of pAS ranged from 0.672 mg/ml against *B*. *caeruleus* (India) to 0.0297 mg/ml against *N*.*haje* (Egypt) venom. A higher potency value (P) implies a better capability of venom neutralization by the pAS.

**Table 7:** *In vivo* neutralization of 11 homologous elapid venoms by ‘pan-specific’ antiserum.

#	Species	WHOcategory	Challenge dose^a^	LD_50_ (μg/g)	ED_50_^b^	ER_50_ (mg/ml)^c^	P (mg/ml)^d^
1	*N*. *kaouthia* (Thailand)	1	5	0.18	150.00	0.126	0.101
				(0.12–0.27)	(137.07–164.15)	(0.084–0.189)	
2	*N*. *kaouthia* (Malaysia)	1	5	0.90	111.25	0.890	0.712
				(0.59–1.36)	(73.28–168.89)	(0.583–1.345)	
3	*N*. *kaouthia* (Vietnam)	1	2.5	0.90	111.25	0.465	0.279
				(0.59–1.37)	(73.28–168.89)	(0.305–0.708)	
4	*N*. *philippinensis* (Philippines)	1	2.5	0.18	100.00	0.113	0.068
				(0.12–0.27)	(80.68–123.94)	(0.075–0.169)	
5	*N*. *sputatrix* (Indonesia)	1	2.5	0.90	125.00	0.387	0.232
				(0.59–1.36)	(117.72–132.73)	(0.254–0.585)	
6	*N*. *atra* (China)	1	2.5	0.88	89.89	0.587	0.352
				(0.84–0.91)	(59.21–136.46)	(0.561–0.607)	
7	*N*. *atra* (Taiwan)	1	2.5	0.56	50.00	0.644	0.386
				(0.37–0.84)	(40.34–61.97)	(0.426–0.966)	
8	*B*. *candidus* (Indonesia)	1	5	0.11	37.5	0.352	0.282
				(0.07–0.17)	(34.27–41.04)	(0.224–0.544)	
9	*B*. *candidus* (Northeast Thailand)	1	5	0.09	55.63	0.170	0.136
				(0.06–0.14)	(36.64–84.45)	(0.113–0.264)	
10	*B*. *multicinctus* (China)	1	5	0.014	10.04	0.153	0.123
				(0.010–0.021)	(9.55–10.55)	(0.110–0.230)	
11	*B*. *multicinctus* (Taiwan)	1	5	0.028	19.57	0.172	0.137
				(0.018–0.042)	(15.99–23.95)	(0.110–0.258)	

^a^ Challenge dose corresponds to the number of venom LD_50_s used per animal.

^b^ ED_50_: Median Effective Dose: volume of antiserum required to protect half of the mice injected with the corresponding challenge dose of venom.

^c^ ER_50_: Median effective ratio: Ratio of mg venom / ml antivenom in which half of the injected mice are protected.

^d^ P: Potency of antiserum: the amount of venom (mg) that is completely neutralized by a unit volume of antiserum (one ml).

**Table 8:** *In vivo* neutralization of 16 heterologous elapid venoms by ‘pan-specific’ antiserum.

#	Species	WHO category	Challenge dose^a^	LD_50_ (μg/g)	ED_50_^b^	ER_50_ (mg/ml)^c^	P (mg/ml)^d^
1	*N*. *oxiana* (Pakistan)	1	2.5	0.90	60.43	0.875	0.525
				(0.59–1.37)	(52.39–69.70)	(0.574–1.332)	
2	*N*. *sumatrana* (Malaysia)	1	5	0.50	100.00	0.55	0.44
				(0.40–0.62)	(80.68–123.94)	(0.44–0.682)	
3	*N*. *siamensis* (Thailand)	1	5	0.28	178.47	0.177	0.141
				(0.18–0.42)	(161.28–197.49)	(0.113–0.265)	
4	*N*. *naja* (India)	1	1.5	1.80	141.36	0.420	0.140
				(1.18–2.73)	(108.22–184.63)	(0.275–0.637)	
5	*N*. *naja* (Sri Lanka)	1	1.5	1.71	141.36	0.399	0.133
				(1.55–1.88)	(108.22–184.63)	(0.362–0.439)	
6	*N*. *naja* (Pakistan)	1	5	0.30	175.00	0.189	0.151
				(0.27–0.33)	(167.55–182.78)	(0.170–0.257)	
7	*N*. *haje* (Egypt)	1	2.5	0.09	100.00	0.0495	0.0297
				(0.05–0.14)	(80.68–123.94)	(0.0275–0.077)	
8	*N*. *nigricollis* (Cameroon)	1	1.5	0.75	156.57	0.172	0.057
				(0.69–0.82)	(127.95–191.59)	(0.159–0.189)	
9	*B*. *caeruleus* (Pakistan)	1	5	0.05	15.11	0.364	0.291
				(0.04–0.06)	(13.10–17.43)	(0.291–0.437)	
10	*B*. *caeruleus* (Sri Lanka)	1	5	0.06	8.83	0.713	0.571
				(0.04–0.08)	(6.76–11.54)	(0.476–0.951)	
11	*B*. *caeruleus* (India)	1	5	0.10	12.50	0.840	0.672
				(0.08–0.12)	(10.09–15.49)	(0.672–1.008)	
12	*B*. *fasciatus* (Thailand)	2	1.5	1.50	91.24	0.543	0.181
				(1.21–1.86)	(58.29–142.8)	(0.438–0.673)	
13	*B*. *sindanus* (Pakistan)	1	5	0.018	11.41	0.174	0.139
				(0.012–0.027)	(7.29–17.85)	(0.116–0.260)	
14	*O*. *hannah* (Malaysia)	2	2.5	0.90	111.25	0.425	0.255
				(0.59–1.36)	(73.28–168.89)	(0.278–0.642)	
15	*N*. *melanoleuca* (North Cameron)	1	5	0.33	141.36	0.268	0.215
				(0.22–0.51)	(108.22–184.63)	(0.179–0.415)	
16	*N*. *nubiae* (Egypt)	2	2.5	0.28	78.29	0.197	0.118
				(0.22–0.37)	(63.98–95.80)	(0.155–0.260)	

^a^ Challenge dose corresponds to the number of venom LD_50_s used per animal.

^b^ ED_50_: Median Effective Dose: volume of antiserum required to protect half of the mice injected with the corresponding challenge dose of venom.

^c^ ER_50_: Median effective ratio: Ratio of mg venom / ml antivenom in which half of the injected mice are protected.

^d^ P: Potency of antiserum: the amount of venom (mg) that is completely neutralized by a unit volume of antiserum (one ml).

There were 4 heterologous venoms *(B*. *fasciatus*, *N*. *nigricollis* (Cameroon), *N*. *naja* (India) and *N*. *naja* (Sri Lanka)) that were neutralized by pAS only when the venom challenge dose were low at 1.5LD_50._ At this venom dose in the absence of pAS, all the mice died.

## Discussion

In the present study, a novel yet simple approach was used to produce a pan-specific snake antiserum with wide paraspecificity. Nine toxin fractions (TFs) derived from various elapid venoms together with other three crude venoms were used at very low dose as immunogen. The use of TFs from 9 elapid venoms as immunogen reduced the amount of high MW, highly immunogenic and largely non-toxic venom proteins in the immunogen. The low doses of TFs/venoms not only reduced the possible toxicity on the horse but could induce high affinity neutralizing antibody [43]. The horse antiserum obtained showed wide paraspecificity and neutralized the lethality of 27 homologous and heterologous elapid venoms from various countries of Asia including 4 from Africa.

There are a few methods whereby high MW venom proteins can be selectively removed from the venom. Since the elapid 3FTs (neurotoxins and cytotoxins) and phospholipase A_2_ are relatively heat-stable at acidic pHs, heating of elapid venoms at 100°C at pH 5.0 could quantitatively recover the α-neurotoxin, cytotoxins and phospholipase A_2_ of *N*. *kaouthia* venom [44]. However, this method may not be applicable to the venoms of *Bungarus spp*. because the presynaptic neurotoxins (toxic phospholipases A_2_) may not be able to withstand high temperature. Alternatively, separation of the high MW venom proteins from the toxic components could also be achieved by size-exclusion chromatography. In this work, we chose to use ultra-filtration method as the method is simpler, faster, economical as well as effective. This method works by filtering proteins below a certain molecular weight through a non-denaturing polyethersulfone membrane with specific pore size selected prior to ultrafiltration. TFs of lower MW hence could be optimally recovered from the filtrate and was devoid of high MW proteins as shown by SDS-PAGE (Fig 1 and S1 Fig). The amount of the high MW venom proteins removed by ultra-filtration ranged from 4.7% for *B*. *multicinctus* (China) venom to 17.52% for *N*. *kaouthia* (Vietnam) venom. Though relatively small in quantity, these high MW proteins are highly immunogenic and have been shown by Western Blot to induce high titer of antibody [45]. Although ‘antigenic competition’ [46] in antivenom production has not yet been established, the presence of these proteins in the immunogen mix could possibly interfere with the production of antibodies specific against the lethal toxins of the venom as have been observed with other venoms [47–49].

In this study, the venomic method of comparing the proteomes of TF and crude venom of *N*. *kaouthia* provided evidence that all the important toxins of the venom were recovered in the TF. Also, LD_50’s_ of the crude venoms and the TFs of the 4 selected venoms were found to be comparable, further confirming that essentially all the principal toxic components of the venom were present in the respective TF. The results support the use of TFs as immunogen in the preparation of antiserum, as the TFs contain essentially all the lethal components that should be neutralized for effective treatment.

With the ‘low dose, low volume multi-site immunization protocol’ employed [36, 37], the sera antibody ELISA titers of all 3 horses rose rapidly and reached plateau in only about 4–6 weeks. The fast kinetics of antibody production, observed previously with elapid and viperid venoms [36, 37, 42], could significantly reduce the cost of antivenom production. However, with the many and heterogeneous venom proteins used as immunogens and as ELISA antigens of this study, the maximum ELISA titers varied widely. It seemed that, for the homologous venoms, the titers of the pAS against the *Naja* venoms were higher than those against the *Bungarus* venoms. When compared with the *in vivo* neutralization results, the ELISA results did not seem to correlate well with the *in vivo* neutralization results. Thus, the ELISA titers could only be used as a rough guide on the kinetics of antibody production but not on the neutralization capacity of the sera antibody. The reason behind this observation is not apparent at the moment. The relative content of the lethal toxins in the TF proteins is likely to be involved in this correlation. For example, while all the members of the 3FTs (postsynaptic neurotoxins, cytotoxins, weak neurotoxins) could act as antigens and contribute to the ELISA titers, but only the postsynaptic neurotoxins, short and long, play major parts in the lethality of the venom and in the neutralization of the pAS.

The TFs used as immunogens were derived from venoms of WHO Category 1 medically important snakes of several countries in Asia, so were the three venoms used. Moreover, some species were taken from different regions or countries. The reason for this was that intra-specific variations in venom compositions and clinical manifestations have been widely observed [50–54] and this variation could increase the diversity of the venom toxins and enhance the snake coverage of the resulting antiserum. For example, *N*. *kaouthia* venoms were from Thailand, Vietnam and Malaysia; these venoms had been shown to exhibit intra-specific variations in toxin profiles and lethality [34]: *N*. *kaouthia* venom from Thailand is high in long neurotoxin, the venom from Malaysia is low in neurotoxin but very high in cytotoxin, whereas venom from Thailand is high in weak neurotoxin; and both venoms from Malaysia and Vietnam contain more short neurotoxins. *N*. *philippinensis* and *N*. *atra* venoms are both known to contain very high content of short neurotoxin [55, 56]. *N*. *sputatrix* venom also contained mainly short neurotoxin, large amount of cytotoxin and lethal PLA_2_ [57]. Thus, together, the *Naja* venoms selected contain a good balance of 3FTs, including long, short and weak neurotoxin as well as cytotoxins.

On the other hand, antigens of krait venoms were contributed by *B*. *candidus* and *B*. *multicinctus* venoms to cover the lethal β-neurotoxins that induce presynaptic neurotoxic effect, distinct from the post-synaptic neurotoxicity caused by the cobra venoms. In the case of *B*. *candidus*, the venom was from Bandung (Indonesia), Thailand (northeast and south). We did not have the *B*. *candidus* venom from Malaysia at the time of immunization, *B*. *candidus* venom from southern Thailand was therefore used as a substitute.

Overall, the purpose of this approach (use of very low doses of many TFs/venoms from different geographical areas as immunogen) was to increase the diversity and repertoire of the epitopes of the lethal toxins exposed to the horse, and thereby increasing the paraspecificity of the resulting antiserum. Moreover, the use of very low doses of immunogens was to induce high affinity specific antibodies [43]. Three crude venoms were included as immunogen because these venoms were available in very limited amounts at the time, and were not enough for the entire immunization program if they were prepared as TFs. Whether the small amount of high MW proteins of these 3 crude venoms had any inhibitory effect [47–49] or adjuvant effect [58] on the antibody production observed here is not known.

It is encouraging that the pAs produced using the mixture as immunogen could neutralize all the 27 homologous and heterologous elapid venoms tested albeit with varying potencies. The amount of *B*. *candidus* (South Thailand) venom which was used as immunogen was not available for the *in vivo* neutralization assay. It can be seen that the neutralization potencies (P) which theoretically is independent of the challenge venom dose [40], varied widely. For example, among the homologous venoms, the P value against *N*. *kaouthia* (Thailand) was only 0.101 mg/ml while the P value against *N*. *kaouthia* (Malaysia) was 0.712 mg/ml. The LD_50s_ of the Thai and the Malaysian venoms were 0.18 and 0.90 μg/g mouse, respectively. Moreover, the heterologous *N*. *oxiana* (Pakistan) venom with the LD_50_ of 0.90 was neutralized by pAS with P of 0.525 mg/ml. Thus, it seems that the difference in neutralization potency of pAs against these venoms correlated with the venom lethality which in turn related to the difference in the contents of the lethal postsynaptic neurotoxin of these venoms [34]. However, this might be a simplistic interpretation and the situation may be more complex. The number of epitopes on the lethal toxins and the binding affinity of the pAS antibodies against these epitopes, are likely to play important roles in determining the neutralization potency of the antiserum. Thus, among the heterologous venoms, the LD_50s_ of *B*. *fasciatus* and *B*. *sindanus* were 1.50 and 0.018 μg/g mouse, respectively. The former krait venom was about 83 folds less toxic than the latter, yet the P values of the pAS against these two venoms were quite comparable (Table 8). More detailed information of the biochemistry, immunochemistry and pharmacology of the toxins of these venoms are needed to gain a thorough understanding of the relative neutralization capacity of the pAS against them.

It should be emphasized that the relatively low neutralization potencies against some venoms reported here are those of the crude horse antiserum, and could not be strictly compared with those of the fractionated and concentrated therapeutic IgG or F(ab’)_2_ antivenom. Depending on the manufacturer and the starting potency of the horse sera, the antisera are usually processed and concentrated several folds so that the final antibody product passes the minimal potency requirement. Thus, the pAS prepared in the present study could be successfully processed into an effective pan-specific antivenom against most of the medically important cobra and kraits of many parts of Asia.

It is interesting to note that the pAS could also neutralize the venoms of 4 African cobra: *N*. *melanoleuca* (North Cameroon), *N*. *nubiae* (Egypt), *N*. *nigricollis* (Cameroon) and *N*. *haje* (Egypt), though with the neutralization potencies not quite as high as those against the homologous Asiatic cobras. Inclusion of these venoms (as TFs) in the immunogen mix could possibly provide better neutralization against these and possibly some other African elapid venoms as well.

As discussed above, at this stage we could not compare the *potencies* of our antiserum directly to the *potencies* of commercial antivenoms available in the region. However, the commercial antivenoms available in this region are usually country-specific and are raised against only limited species important to the country; antivenoms produced as such generally have limited therapeutic value for use against other species from another region/country, due to vast variations affected by geographical and inter-species factors. These variations were believed to be taken care of by the unique method innovated in this study.

The current study thus demonstrated the feasibility of producing a pan-specific antivenom against many cobras and kraits in Asia through a novel yet simple immunization strategy. The procedure involved simple and inexpensive ultrafiltration method and small quantities (about 5 mg/venom) of the homologous venoms for the entire immunization program. It could readily be applied to the production of any pan-specific AS against elapid (*Naja* and *Bungarus*) snakes. The findings should provide useful insights into the optimization of immunogen preparation with the aim to broaden the paraspecificity of antivenom for clinical use, an effort in line with the Global Snakebite Initiative [59]. With the economy of scale, the pan-specific antivenom could be produced economically and offered as a more sustainable and affordable supply to many countries; hence saving the lives of many victims succumbed to snakebite envenomation.

It is also hoped that the simple ‘low dose, diverse toxin repertoires’ strategy employed in this study could be adapted in the preparation of pan-specific antisera against medically important elapids of other continents.

## Supporting Information

S1 Fig SDS-PAGE of crude venoms and TFs together with the relative abundance of various elapid venom proteins. S1 A, *N*. *kaouthia* (Malaysia); S1 B, *N*. *kaouthia* (Vietnam); S1 C, *N*. *sputatrix* (Indonesia); S1 D, *N*. *atra* (China); S1 E, *N*. *atra* (Taiwan); S1 F, *B*. *candidus* (Indonesia); S1 G *B*. *candidus* (Northeast Thailand); S1 H, *B*. *multicinctus* (China); S1 I, *B*.*multicinctus* (Taiwan).
(TIF)

S2 Fig Kinetics of ELISA antibody response against TFs of various homologous elapid venoms. The ELISA titers against the TFs are shown: S2 A, *N*. *kaouthia* (Thailand); S2 B, Pure NTX3 of *N*. *kaouthia* (Thailand); S2 C, *N*. *philippinensis* (The Philippines); S2 D, *N*. *sputatrix* (Indonesia); S2 E, *N*. *atra* (China); S2 F, *N*. *atra* (Taiwan); S2 G, *B*. *candidus* (Indonesia); S2 H, *B*. *multicinctus* (China).
(TIF)

S3 Fig Kinetics of ELISA antibody response against TFs of various heterologous elapid venoms. The ELISA titers against the TFs are shown: S3 A, *O*. *hannah* (Thailand), S3 B, *N*. *siamensis* (Thailand); S3 C, *N*. *sumatrana* (Malaysia); S3 D, *N*. *oxiana* (Pakistan); S3 E, *N*. *naja* (India); S3F, *B*. *caeruleus* (India); S3 G, *B*. *sindanus* (Pakistan).
(TIF)
