# Supplementary figures and images for: A Simple and Novel Strategy for the Production of a Pan-specific Antiserum against Elapid Snakes of Asia
Source: PLoS Negl Trop Dis. 2016 Apr 8;10(4):e0004565. doi: 10.1371/journal.pntd.0004565 (PMC4825939; doi:10.1371/journal.pntd.0004565)

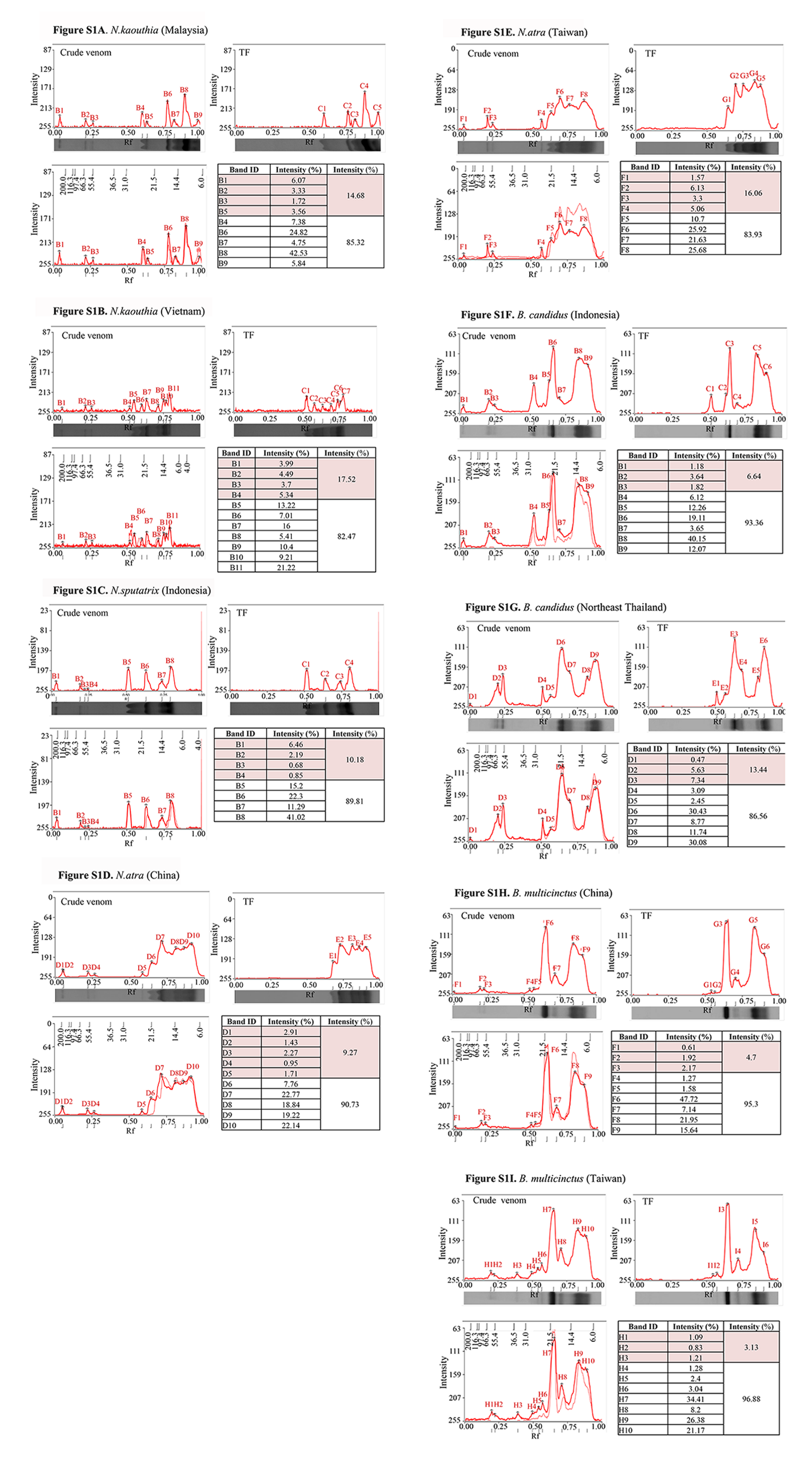

Supplement: S1 Fig — S1 A, N. kaouthia (Malaysia); S1 B, N. kaouthia (Vietnam); S1 C, N. sputatrix (Indonesia); S1 D, N. atra (China); S1 E, N. atra (Taiwan); S1 F, B. candidus (Indonesia); S1 G B. candidus (Northeast Thailand); S1 H, B. multicinctus (China); S1 I, B.multicinctus (Taiwan). (TIF) [file pntd.0004565.s001.tif]

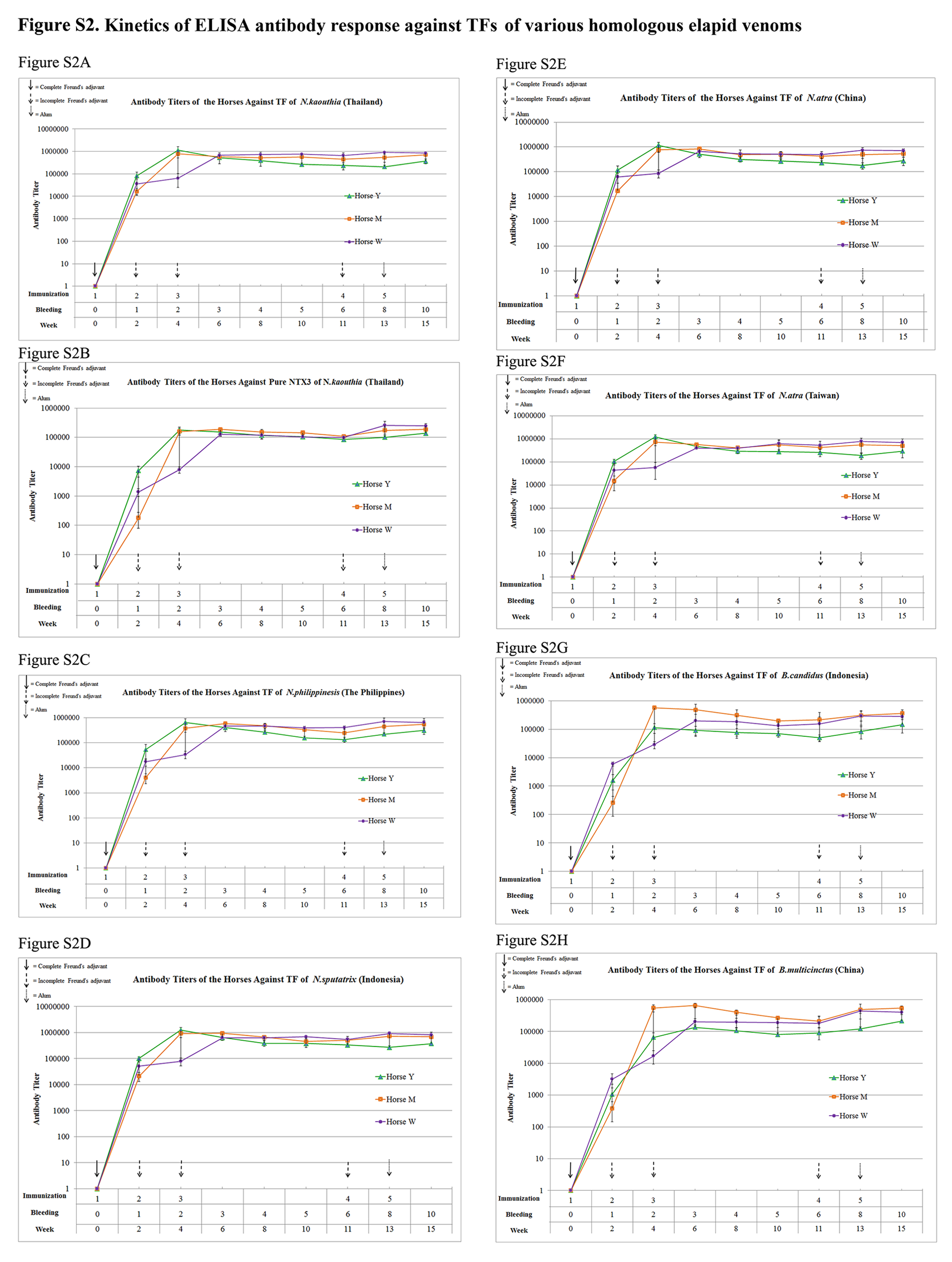

Supplement: S2 Fig — The ELISA titers against the TFs are shown: S2 A, N. kaouthia (Thailand); S2 B, Pure NTX3 of N. kaouthia (Thailand); S2 C, N. philippinensis (The Philippines); S2 D, N. sputatrix (Indonesia); S2 E, N. atra (China); S2 F, N. atra (Taiwan); S2 G, B. candidus (Indonesia); S2 H, B. multicinctus (China). (TIF) [file pntd.0004565.s002.tif]

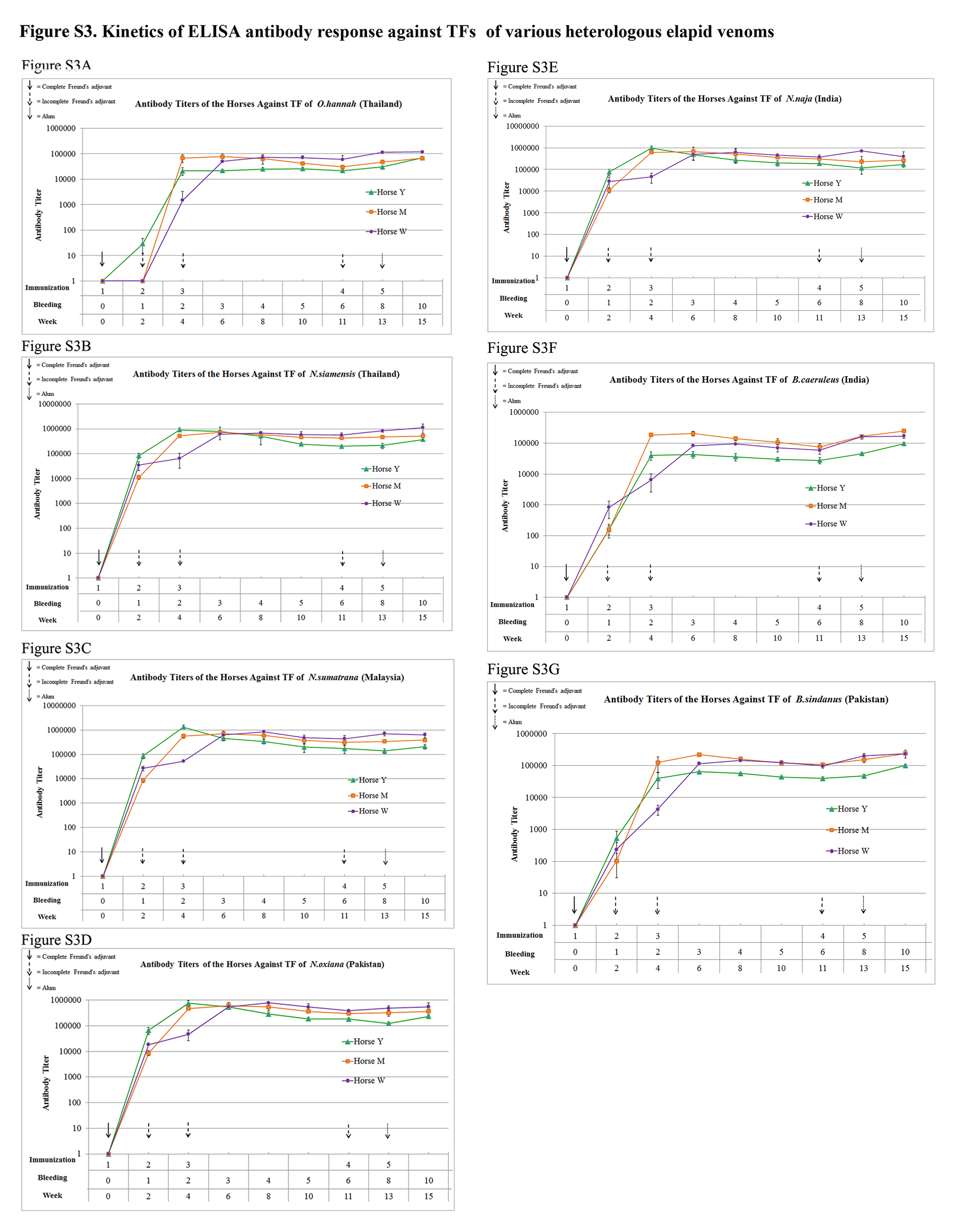

Supplement: S3 Fig — The ELISA titers against the TFs are shown: S3 A, O. hannah (Thailand), S3 B, N. siamensis (Thailand); S3 C, N. sumatrana (Malaysia); S3 D, N. oxiana (Pakistan); S3 E, N. naja (India); S3F, B. caeruleus (India); S3 G, B. sindanus (Pakistan). (TIF) [file pntd.0004565.s003.tif]
